# Annual Circular of the Massachusetts Medical College

**Published:** 1846-09

**Authors:** 


*Annual Circular of the Massachusetts Medical College, for* 1846–7.

Besides the announcement of the course of instruction for 1846–7, in this old and distinguished medical institution, which will commence its next session under advantages very superior to any which it has heretofore enjoyed, the circular before us contains a brief but interesting history of the College, as well as a catalogue of its graduates.

A new and spacious college edifice is in process of erection, which will be completed in time for the coming course of lectures. It is situated near the Massachusetts General Hospital, where clinical lectures are given three times a week, and which is accessible to students throughout the year.

The facilities offered by this venerable institution for the acquirement of a thorough knowledge of the various branches of general medicine are equal to those of any medical school in the union.—*Balt. Ed.*

